# Role of Mas Receptor Antagonist A799 in Renal Blood Flow Response to Ang 1-7 after Bradykinin Administration in Ovariectomized Estradiol-Treated Rats

**DOI:** 10.1155/2015/801053

**Published:** 2015-09-03

**Authors:** Aghdas Dehghani, Shadan Saberi, Mehdi Nematbakhsh

**Affiliations:** ^1^Water & Electrolytes Research Center, Isfahan University of Medical Sciences, Isfahan 81745, Iran; ^2^Department of Physiology, Isfahan University of Medical Sciences, Isfahan 81745, Iran; ^3^Isfahan Institute of Basic & Applied Sciences Research, Isfahan 81546, Iran

## Abstract

*Background*. The accompanied role of Mas receptor (MasR), bradykinin (BK), and female sex hormone on renal blood flow (RBF) response to angiotensin 1-7 is not well defined. We investigated the role of MasR antagonist (A779) and BK on RBF response to Ang 1-7 infusion in ovariectomized estradiol-treated rats.* Methods*. Ovariectomized Wistar rats received estradiol (OVE) or vehicle (OV) for two weeks. Catheterized animals were subjected to BK and A799 infusion and mean arterial pressure (MAP), RBF, and renal vascular resistance (RVR) responses to Ang 1-7 (0, 100, and 300 ng kg^−1^ min^−1^) were determined.* Results*. Percentage change of RBF (%RBF) in response to Ang1-7 infusion increased in a dose-dependent manner. In the presence of BK, when MasR was not blocked, %RBF response to Ang 1-7 in OVE group was greater than OV group significantly (*P* < 0.05). Infusion of 300 ng kg^−1^ min^−1^ Ang 1-7 increased RBF by 6.9 ± 1.9% in OVE group versus 0.9 ± 1.8% in OV group. However when MasR was blocked, %RBF response to Ang 1-7 in OV group was greater than OVE group insignificantly.* Conclusion*. Coadministration of BK and A779 compared to BK alone increased RBF response to Ang 1-7 in vehicle treated rats. Such observation was not seen in estradiol treated rats.

## 1. Introduction

Women are shown to be protected against cardiovascular and renal diseases before menopause, suggesting that estrogen has beneficial roles in this respect [1–3]. Estrogen affects renin-angiotensin system (RAS) by stimulating the depressor pathway of this system [4–6], while this sex hormone enhances the kallikrein-kinin system (KKS), leading to the renoprotective effect [7, 8].

The heptapeptide angiotensin 1-7 (Ang 1-7) is a biologically active peptide of RAS, especially in the kidney [9, 10] via G-protein coupled Mas receptor (MasR) [11]. Ang 1-7 induces vasodilator response to attenuate the actions of angiotensin II (Ang II) [12] and therefore plays pivotal role in cardiovascular and renal systems [13, 14]. Endogenous levels of Ang 1-7 are increased by angiotensin converting enzyme (ACE) or Ang II type 1 receptor (AT1R) inhibitors indicating that the protective effects of ACE or AT1R blockers are exerted by augmenting of this peptide [15, 16]. Ang 1-7 formation is different in the circulation and kidney [17, 18]. In circulation, neutral endopeptidase (NEP) is the main enzyme that produces Ang 1-7 [19], while the major enzyme that contributes to renal Ang 1-7 synthesis is ACE2 as ACE homologue [17, 18]. In general, the ACE2/Ang 1-7/Mas axis has a key role in renal hemodynamics.

KKS is also involved in the control of renal hemodynamics and function [20]. This system forms active peptides called kinins that have two distinct receptors, namely, B1 and B2 receptors [21]. Bradykinin (BK) is a main component of the KKS generated from kininogens by the kallikreins [21] and is expressed within the kidney [22]. In the kidney, the local infusion of BK enhances sodium and water excretion [23], while it mediates some functions via B2 receptor, including vasodilatation, natriuresis, and dieresis [23, 24]. It is well known that ACE participates in degradation of BK, and ACE inhibitor elevates the BK level. Therefore, the positive effects of ACE inhibitor in the kidney can be exerted by enhancing and prolonging the effects of BK [25, 26]. Ang 1-7 increases BK-induced vasodilator effects by activation of mediated factors, nitric oxide (NO) and prostaglandin release [21, 27]. It is demonstrated that cooperation between Ang 1-7 and BK potentiates vasodilatory response, mediated by endothelium-dependent release of NO [27], and coadministration of Ang 1-7 and BK causes hypotension [28]. In addition, B2 receptor antagonist, HOE-140, abolishes the vasodepressor effects of Ang 1-7 [28]. On the other hand, inhibition of MasR by A779 leads to blockade of BK and potentiating activity of Ang 1-7, indicating that MasR is involved in mediating the vasodilation effect [29].

Renal diseases usually are influenced by RBF disturbance [30–32] hormone dependently [3], and MasR, BK, and NO are abundant in the kidney too [33–36]. On the other hand, female sex hormone, estrogen, plays an important role in regulation of RAS and KKS [5–7]. This complex data raises the question about the role of estrogen and BK in renal Ang 1-7-Mas axis function.

Therefore, it is hypothesized that BK and estradiol may influence RBF response to Ang 1-7 via MasR. To examine this hypothesis, MasR was inhibited by A779 and in the presence of BK, the response of RBF to Ang 1-7 was measured in ovariectomized rats treated with estradiol, and the results were compared with those obtained from the control group.

## 2. Methods and Materials

### 2.1. Animals

Female rats were housed in the animal room with controlled temperature of 23–25°C and 12 h light/dark cycle. The rats were kept in cages with free access to water and chow. All experimental procedures were in advance confirmed by Isfahan University of Medical Sciences Ethics Committee.

Female Wistar rats (200 ± 20 g) were anesthetized (0.06 g/kg of ketamine 10% and xylazine 2% solution) and ovariectomized as described before [37]. After one week of recovery, the animals were randomly assigned into two groups named OVE as the treated group and OV as the control group. The animals in OVE group received estradiol valerate (500 g/kg/twice weekly, im) dissolved in sesame oil, while OV group received vehicle (sesame oil) for estradiol for two weeks. The animals were then subjected to surgical procedure.

### 2.2. Surgical Procedure

The rats were anesthetized by urethane (1.7 g/kg^−1^ i.p.; Merck, Germany). The trachea was cannulated for air ventilation and polyethylene catheters were inserted into the carotid and femoral arteries and jugular vein for direct mean arterial pressure (MAP) and renal perfusion pressure (RPP) measurements and drug administration, respectively. Arterial catheters were connected to a pressure transducer and bridge amplifier (Scientific Concepts, Melbourne, VIC, Australia) and attached to a data acquisition system. The bladder also was catheterized to collect urine output. Body temperature was monitored continuously through the experiment. An adjustable clamp was put around the aorta above the renal artery to maintain RPP at the control level during Ang 1-7 infusion. With midline incision, the kidney was exposed and placed in a kidney cup. A transit-time ultrasound flow probe (Type 2BS; Transonic Systems, Ithaca, NY, USA) was placed around the renal artery to measure RBF. Then, 30–60 minutes after the equilibration period, basal MAP, RPP, and RBF were recorded and RVR was calculated as RPP/RBF.

### 2.3. Experimental Protocol

Both groups of OV and OVE underwent the experimental protocol according to the following procedure. After measurement of MAP, RPP, and RBF in the equilibration time, A779 or combination of BK and A779 was infused via the vein catheter. Therefore, in each group, we assigned two subgroups. Subgroup 1 and subgroup 2 from OV group received BK and BK + A779, respectively. Such treatments were applied in subgroups 1 and 2 from OVE group. A779 is a selective MasR antagonist and also has negligible affinity for Ang II receptors [38]. It was administrated at bolus doses of 50 *μ*g/kg followed by continuous infusion at 50 *μ*g/kg/h. BK as B2 agonist was infused at 150 *μ*g/kg/h. Both BK and A779 were infused for the entire duration of experiment until the experiment was finished (end of Ang 1-7 infusion).

Thirty minutes after administration of agonist and antagonist, intravenous infusion of Ang 1-7 at graded doses of 0, 100, and 300 ng kg^−1^ min^−1^ was performed using a microsyringe pump (New Era Pump System Inc. Farmingdale, NY, USA). Each dose was given until the response of MAP reached plateau (approx. 15 min). Then, the rats were sacrificed humanely and the left kidney was weighed rapidly. The *t*-Student test was applied to analyze the baseline data and ANOVA for repeated measures was used for other data.

## 3. Results

### 3.1. Baseline Measurement

**Figure 1:**
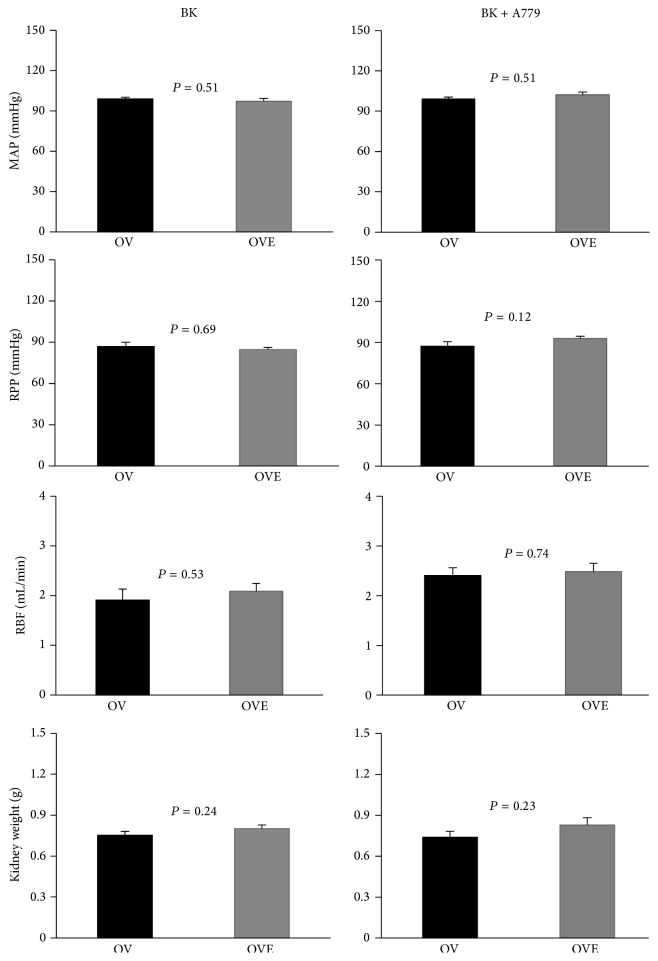
Baseline hemodynamic parameters in BK and BK + A779 groups in ovariectomized untreated and ovariectomized estradiol-treated rats. Data are presented as mean ± SEM. MAP, mean arterial pressure; RPP, renal perfusion pressure; RVR, renal vascular resistance; RBF, renal blood flow* per* gram kidney weight. There were no significant differences between the groups.

In baseline measurements, before infusion of BK or BK + A779, the groups were not significantly different in terms of MAP, RPP, RBF, and RBF per gram kidney weight (Figure 1).

### 3.2. Effect of Agonist and Antagonist

**Figure 2:**
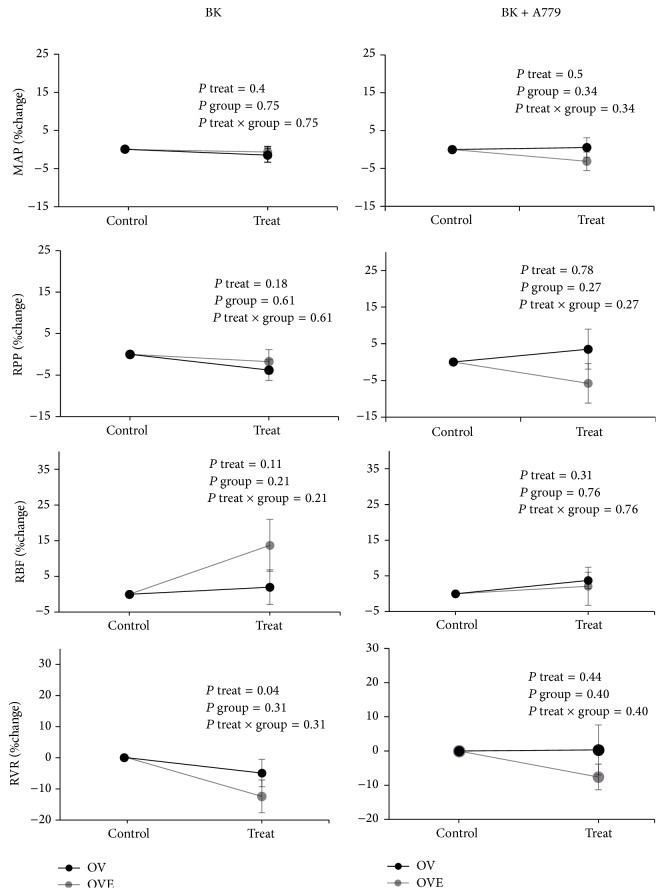
Parameters were recorded in ovariectomized and ovariectomized estradiol-treated rats before and after administration of BK or A779 + BK. Data are presented as mean ± SEM of percentage changes from the baseline. MAP, mean arterial pressure; RPP, renal perfusion pressure; RVR, renal vascular resistance; RBF, renal blood flow* per* gram kidney weight.* P* values were derived from repeated measures ANOVA.

MAP and RPP did not significantly alter when BK or BK + A779 was administered in both OV and OVE groups. The results also indicated that BK or BK + A779 infusion had no significant effect on RBF percentage change (%RBF, Figure 2).

### 3.3. Responses to Ang 1-7 Infusion

**Figure 3:**
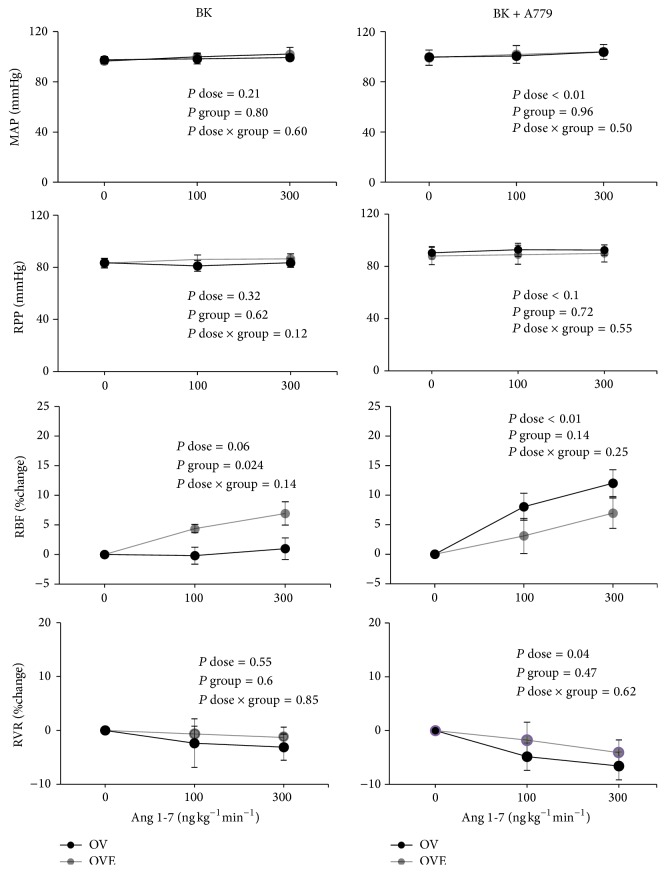
RBF, MAP, RPP, and RVR responses to Ang 1-7 were present when BK or A779 + BK was administrated. Data are shown as mean ± SEM. MAP, mean arterial pressure; RPP, renal perfusion pressure; RVR, renal vascular resistance; RBF, renal blood flow.* P* values were derived from repeated measures ANOVA.

A slight increase of MAP response to Ang 1-7 infusion was observed when both A779 and BK were infused. For example, 300 ng kg^-1 ^min^−1^ Ang 1-7 increased MAP from 99.8 ± 2.4 (0 ng kg^−1^ min^−1^) to 103.7 ± 2.9 mmHg in OV group and from 99.2 ± 6 (0 ng kg^−1^ min^−1^) to 103.8 ± 5.9 mmHg in OVE group. However, in both the OV and OVE groups, the difference of MAP alteration between the two subgroups induced by Ang 1-7 infusion was not statistically meaningful (Figure 3). As mentioned before, RPP remained constant at the basal value during Ang 1-7 infusion. Ang 1-7 infusion increased %RBF in a dose-dependent manner in all the groups (*P* dose < 0.001). BK alone administration increased %RBF response to Ang 1-7 in the OVE group compared with the OV group (*P* < 0.05). For example, 300 ng kg^−1^ min^−1^ Ang 1-7 increased RBF by 6.9 ± 1.9% in OVE group versus 0.9 ± 1.8% in OV group. Interestingly, when BK plus A779 was infused, RBF response was enhanced dose-dependently in both groups with greater response in the OV group. For example, 300 ng kg^−1^ min^−1^ Ang 1-7 increased RBF by 12 ± 2.2% in OV group versus 7 ± 2.5% in OVE group.

## 4. Discussion

The present study was performed to determine the effect of MasR antagonist, A779, on the RBF response to Ang 1-7 in the presence of BK in ovariectomized rats that were treated with vehicle or estradiol. Our major finding is that administration of BK promotes RBF response to Ang 1-7 in estradiol-treated rats when MasR is not blocked. However, surprisingly, when A779 was added, RBF response to Ang 1-7 increased in a dose-dependent manner in both the OV and OVE groups, while RBF response in the OV group was insignificantly greater than that in the OVE group.

Some observations show that estrogen contributes to modulate components of renal KKS and RAS, and it regulates renal hemodynamics via these two systems [6–8, 39]. B2 receptor mRNA levels were reduced by ovariectomy in the aorta and kidney, and this alteration is reformed by estrogen replacement [7]. Animal study also supports that renal kallikrein and kallikrein mRNA levels of rats are enhanced in females compared with males; and these factors were decreased by ovariectomy and estrogen treatment returns them back to normal levels [39]. Additionally, hormone replacement therapy (HRT) in postmenopausal women increases plasma concentration of BK and decreases ACE activity [40]. Estradiol enhances expression of renal ACE2 and consequently increases Ang 1-7 level [6]. Previous study showed that, in the heart of ovariectomized rats, estrogen depletion does not alter MasR expression, but estradiol therapy reduces its expression [41]. Estrogen replacement also enhances the relaxation response of Ang 1-7 in female ovariectomized rats, and this alternation is blocked by Ang 1-7 receptor antagonist, D-[Ala^7^]-Ang 1-7 [42]. In the uterine arteries of sheep, estrogen stimulates the vasodilator response to BK by enhancement of NO release and NO synthase (NOS) activity [43]. Therefore, renoprotective role of estrogen may result from enhanced plasma levels of NO, BK, and Ang 1-7 and reduction in the arterial blood pressure and ACE activity. Our results are in accordance with the findings of the studies that declare that in the presence of BK estradiol enhances the RBF response to Ang 1-7.

The kidney effects of Ang 1-7 and BK are a complex pathway. Ang 1-7 regulates renal hemodynamics, glomerular filtration rate, and tubular transport [44–47]. It also modifies RBF through release of prostanoids and NO [47–50], where Ang 1-7 via MasR regulates renal function [51] in a gender dependent manner [34]. It is reported that Ang 1-7 increases blood flow in the kidney, brain, and mesentery [48]. Nematbakhsh and Safari suggested that RBF response to Ang 1-7 is different between males and females [34] possibly due to higher MasR expression in females [52]. This is while BK exerts variable effects on the renal vascular bed. Ren et al. demonstrated that BK has a vasodilator effect on efferent arterioles [53], and its effect on vascular tone is modulated by releasing vasodilator mediators such as NO and prostaglandins [24, 54, 55]. It seems that renal B2 receptor causes vasodilation. Hock et al. confirmed that the selective B2 receptor antagonist, icatibant, inhibits relaxation response to BK in renal vascular preparation [56]. In another study, it was demonstrated that des-Arg9-BK, a selective B1 receptor agonist, was involved in vasoconstriction in the kidney and this response is abolished by B1 receptor antagonist [57]. In fact, the main renal vascular response to BK in the isolated perfused kidney was vasodilation [58]. The dose of BK is also another factor contributing to different actions of BK on renal circulation. BK in high doses produces vasoconstriction in renal vessels, while at low doses it causes vasodilation [59]. Hoagland et al. observed that intravenous administration of BK induces significant increase in RBF and decrease in RVR [33]. In addition, BK increases medullary RBF but does not alter cortical RBF [55]. Our data supports that BK enhances RBF in response to Ang 1-7 in a dose-dependent manner. It is reported that, in B2 receptor knockout mice, RBF and blood pressure do not alter compared with the control mice. However, when the two groups were subjected to high salt diet, RBF decreased in B2 receptor knockout mice [60]. Therefore, the regulatory action of endogenous BK on renal function and blood pressure is dependent on physiological and pharmacological conditions [60].

Another point is the MasR effects. An in vivo experiment showed that Ang 1-7 induces vasodilation of mesenteric vessels by facilitation of BK, while losartan, as an AT1R blocker, does not affect this mechanism. However, A779 blocks this vasodilation, suggesting that BK-potentiating activity of Ang 1-7 is not dependent on its interaction with AT1R while MasR is involved [29]. MasR deficient vessels show vascular endothelium dysfunction. Peiró et al. evaluated response to Ang 1-7 in Mas deficient vessels compared with wild type mice treated with A799 [61]. According to the results, Ang 1-7 mediated relaxation was reduced by 40% in isolated vessels of knockout MasR; and A799 diminished dilation response to Ang 1-7 [61]. Additionally, residual vascular relaxation was demonstrated in response to Ang 1-7; therefore, this peptide may act via another pathway [62]. In another study, mesenteric vessels were isolated and A779 and NO synthase (NOS) inhibitor, L-NAME, were administrated and it was shown that the vasorelaxant responses of Ang 1-7 and BK were abolished by A779 and L-NAME [63]. It is concluded that MasR has a central role in endothelial NO-mediated relaxation by Ang 1-7 and BK [63]. In the current study, not only did blockade of MasR significantly enhance RBF response to Ang 1-7, but also this RBF alternation is higher in the vehicle group than in the estradiol-treated animals. Consequently, when MasR is blocked, RBF response to Ang 1-7 may be related to another pathway. We cannot provide the exact mechanism for such observation, but one explanation is that BK potentiates vasodilatory effect of Ang 1-7 by its receptor and also other angiotensin receptors such as AT2R may be involved [64]. To summarize, RBF response to Ang 1-7 seems to be related to several factors including RAS receptors, BK, NO, and prostaglandins. All those factors integrate to control renal hemodynamics. In the absence of one factor, others help compensate and make a greater response.

## 5. Conclusion

Some data support the exclusive MasR mediated vasodilator response of Ang 1-7 [29, 63], while others confirm that other receptors are involved and MasR has a partial role [61, 62]. Furthermore, cooperation between BK and Ang 1-7 appears to have a critical role in the vasodilatory effect that is mediated by BK receptor. Considering the role of estrogen on RAS and KKS, we concluded that BK and estradiol increased RBF response to Ang 1-7 infusion and this action may not be related to MasR.
